# Erectile Dysfunction: A Comprehensive Review of Pathophysiology, Diagnosis and Contemporary Management

**DOI:** 10.3390/medicina62050854

**Published:** 2026-04-30

**Authors:** Felice Crocetto, Ugo Amicuzi, Michele Musone, Ciro Imbimbo, Simone Tammaro, Luigi Napolitano, Pasquale Reccia, Luigi De Luca, Francesco Del Giudice, Marco Stizzo, Michelangelo Olivetta, Dario Di Lieto, Michele Di Mauro, Gennaro Mattiello, Giacomo Puca, Giampiero Della Rosa, Marco Magliocchetti, Michele Giugliano, Raffaele Capoluongo, Mariano Coppola, Silvestro Imperatore, Antonio Madonna, Federico Capone, Dario Del Biondo, Biagio Barone

**Affiliations:** 1 Urology Unit, Department of Neurosciences, Reproductive Sciences and Odontostomatology, Federico II University, 80131 Naples, Italy; felice.crocetto@gmail.com (F.C.); drmichelemusone@gmail.com (M.M.); ciro.imbimbo@unina.it (C.I.); nluigi89@libero.it (L.N.); dario.dilieto@outlook.it (D.D.L.); giacomopuca40@gmail.com (G.P.); marcomag7@outlook.it (M.M.); michele.giugliano@unina.it (M.G.); capoluongoraffaele8@gmail.com (R.C.); marianocoppola99@icloud.com (M.C.); silvestro.imperatore97@gmail.com (S.I.); antoniomadonna1997@icloud.com (A.M.); fedecapone@outlook.it (F.C.); 2 Urology Unit, Department of Woman, Child and General and Specialized Surgery, University of Campania “Luigi Vanvitelli”, 80131 Naples, Italy; simone.tammaro95@gmail.com (S.T.); marcostizzo@hotmail.com (M.S.); giampierodellarosa@gmail.com (G.D.R.); 3 Urology Unit, Azienda Ospedaliera di Rilievo Nazionale Ospedali dei Colli, Monaldi Hospital, 80131 Naples, Italy; reccia.pasquale1@gmail.com; 4 Division of Urology, Department of Surgical Multispecialty, Azienda Ospedaliera di Rilievo Nazionale Antonio Cardarelli, 80131 Naples, Italy; luigideluca86@gmail.com; 5 Department of Urology, University Sapienza, 00185 Rome, Italy; francesco.delgiudice@uniroma1.it; 6 Division of Urology, Department of Surgical Sciences, Azienda Ospedaliera di Rilievo Nazionale Sant’Anna e San Sebastiano, 81100 Caserta, Italy; olivetta.drmichelangelo@gmail.com (M.O.); drmattiellogennaro@gmail.com (G.M.); 7 U.O.C. Anestesia, Rianimazione e Terapia Intensiva, Azienda Ospedaliera di Rilievo Nazionale Ospedali dei Colli, Cotugno Hospital, 80131 Naples, Italy; m.dimauro90@gmail.com; 8 Department of Urology, Ospedale San Paolo, ASL NA1 Centro, 80125 Naples, Italy; dario.delbiondo@aslnapoli1centro.it (D.D.B.); biagio.barone@aslnapoli1centro.it (B.B.)

**Keywords:** erectile dysfunction, PDE5 inhibitors, psychosexual therapy, regenerative therapy, penile prosthesis, testosterone

## Abstract

Erectile dysfunction (ED) is a common multifactorial condition with significant physical, psychological and relational consequences. While historically associated with aging, its rising prevalence among younger men underscores the need for updated diagnostic and therapeutic frameworks. This narrative review synthesizes contemporary evidence on the pathophysiology, diagnostic workup and management of ED, with emphasis on guideline-directed care and emerging treatment modalities. A comprehensive literature search was conducted, with evidence synthesized from key clinical guidelines, landmark trials and recent peer-reviewed studies. Lifestyle optimization remains the foundational step, followed by first-line pharmacotherapy with phosphodiesterase type 5 inhibitors (PDE5is), which demonstrate high efficacy and safety across diverse patient populations. For patients with inadequate PDE5is response, second-line options include alprostadil (intracavernosal, with approximately 70% success rates or intraurethral), vacuum erection devices and penile prosthesis surgery, with patient and partner satisfaction exceeding 95% for the latter when performed in experienced centers. Psychosexual therapy is an integral adjunct, particularly in psychogenic or mixed etiologies. Regenerative approaches such as low-intensity extracorporeal shockwave therapy (Li-SWT) and platelet-rich plasma (PRP) injections are under investigation; current evidence supports their use only in experimental settings due to limited long-term data. A multidisciplinary, individualized strategy—incorporating pharmacologic, surgical and psychosocial interventions—remains the cornerstone of modern ED management. This review critically distinguishes well-established evidence from ongoing clinical debates and translates findings into practical guidance for daily practice. Ongoing technological advances may further refine diagnostic accuracy and treatment personalization, but high-quality studies are needed to establish the role of regenerative and digital tools.

## 1. Introduction

ED is a common multifactorial condition with significant physical, psychological and relational consequences [1,2]. While historically associated with aging, its rising prevalence among younger men underscores the need for updated diagnostic and therapeutic frameworks [3]. The scientific understanding of ED has evolved substantially, recognizing it as a disorder encompassing vascular, neurogenic, hormonal and psychogenic components, frequently coexisting with diabetes, hypertension, obesity, metabolic syndrome and cardiovascular disease [4]. Despite these advances, several key gaps remain. First, the rising prevalence of ED among younger men is poorly characterized and its role as an early cardiovascular sentinel is underutilized in clinical practice [5]. Second, up to one-third of men do not respond adequately to first-line PDE5is, yet standardized management algorithms for non-responders are lacking [6]. Third, regenerative therapies such as Li-SWT and PRP have gained widespread commercial availability without robust evidence supporting their efficacy or long-term safety, creating confusion for clinicians and patients alike [7]. Fourth, existing reviews often describe treatments without critically analyzing evidence quality or translating findings into actionable clinical guidance. To address these gaps, this narrative review synthesizes contemporary evidence on ED pathophysiology, diagnosis and management with a focus on guideline-directed care, PDE5is non-response and critical evaluation of emerging therapies. This review is organized around a single clinical axis: distinguishing what is established from what remains debated in ED management and translating both into actionable guidance for daily practice. For each major topic—pathophysiology, diagnosis, pharmacotherapy, regenerative therapies and surgery—we present evidence-based consensus, ongoing controversies with balanced interpretation, and practical clinical takeaways. Our goal is to provide urologists and primary care physicians with a framework that clarifies decision-making where evidence is strong and navigates uncertainty where it is not.

## 2. Methods

A comprehensive literature search was performed in PubMed, Scopus and Web of Science for articles published between January 2000 and November 2025. The search strategy combined the following key terms: “erectile dysfunction”, “phosphodiesterase type 5 inhibitors”, “testosterone”, “penile prosthesis”, “low-intensity shockwave therapy”, “platelet-rich plasma”, “psychosexual therapy” and “guidelines”. Only English-language articles were considered, including clinical guidelines, randomized controlled trials, systematic reviews, meta-analyses and high-quality observational studies. We excluded case reports, editorials, non-peer-reviewed articles and studies focused exclusively on rare or pediatric causes of ED, as they fell outside the clinical scope of this review. Reference lists of retrieved articles were also screened to identify additional relevant publications. The selection of evidence was based on relevance to the clinical topics covered, with priority given to recent guideline documents and landmark studies. When studies disagreed, we prioritized higher-quality evidence and explicitly noted the disagreement in the text, presenting both sides with a balanced clinical interpretation. Data synthesis followed a narrative approach, structuring the findings according to the key domains of pathophysiology, diagnostic evaluation and stepwise management. To enhance clinical utility, we identified areas of consensus versus controversy. Where multiple randomized trials and guidelines converge, we present findings as established. Where evidence is conflicting or insufficient, we explicitly note ongoing debates and provide balanced clinical guidance based on expert consensus and the authors’ interpretation of available data. All authors participated in the evidence synthesis; disagreements in interpretation were resolved through group discussion until consensus was reached.

## 3. Background and Pathophysiology

ED is a prevalent medical condition among men, defined as the persistent inability to achieve or maintain an erection sufficient for satisfactory sexual performance [1,2]. Although historically associated with the aging process, recent epidemiological studies have documented an increasing prevalence among younger men—a phenomenon that is likely underreported due to patient reluctance to discuss the condition with healthcare providers [8,9]. The scientific understanding of ED has evolved substantially in recent years, with its recognition as a multifactorial disorder encompassing vascular, neurogenic, hormonal and psychogenic components. ED frequently coexists with systemic diseases, including diabetes mellitus, hypertension, obesity, metabolic syndrome and cardiovascular disease [10,11]. Over the past two decades, advances in molecular biology and neurovascular physiology have significantly enhanced our comprehension of the mechanisms underlying penile erection, consequently transforming diagnostic and therapeutic paradigms [12]. The erectile process depends upon a precise physiological balance between psychogenic stimulation, autonomic and somatic neural input, endothelial function and cavernosal smooth muscle tone. Disruption in any of these pathways may precipitate ED [13]. The erectile cascade is initiated by sexual or psychogenic stimulation, which activates the parasympathetic nervous system via the pelvic splanchnic and pudendal nerves. This neural activation stimulates non-adrenergic, non-cholinergic (NANC) neurons and endothelial cells to release nitric oxide (NO) [14]. Subsequently, NO diffuses into adjacent smooth muscle cells and activates guanylate cyclase, leading to increased intracellular concentrations of cyclic guanosine monophosphate (cGMP). The resultant reduction in intracellular calcium concentrations induces relaxation of the cavernosal smooth muscle [15]. Phosphodiesterase type 5 (PDE5) enzymes serve a critical regulatory function by metabolizing cGMP, thereby modulating the duration and intensity of the erectile response and maintaining vascular homeostasis [16]. When endothelial dysfunction reduces NO bioavailability or when smooth muscle exhibits diminished sensitivity to cGMP, erection quality becomes compromised. This physiological mechanism explains the well-established association between ED and chronic conditions that impair endothelial function, such as atherosclerosis, diabetes mellitus and tobacco use [17]. Beyond the vascular and neural mechanisms described above, the pelvic floor musculature—specifically the ischiocavernosus and bulbocavernosus muscles—plays a direct role in achieving and sustaining penile rigidity [18]. The ischiocavernosus muscles compress the crura of the corpora cavernosa, while the bulbocavernosus muscle compresses the bulbar urethra and the corpus spongiosum [12]. Their rhythmic, voluntary and reflex contractions during the rigid phase of erection elevate intracavernosal pressure to levels exceeding systolic blood pressure, thereby maximizing rigidity [19]. Impaired function of these muscles, whether due to neurological injury, surgical disruption or disuse, can contribute to erectile dysfunction. Consequently, pelvic floor rehabilitation is increasingly recognized as an adjunctive therapeutic option, particularly in men with post-prostatectomy or psychogenic ED [20,21]. Obesity deserves specific attention as an independent and modifiable risk factor for ED, given its rising global prevalence and strong mechanistic links to erectile dysfunction [22]. Adipose tissue, particularly visceral fat, functions as an active endocrine organ secreting pro-inflammatory adipokines (e.g., leptin, resistin, tumor necrosis factor-alpha) while reducing anti-inflammatory adiponectin [23]. This adipokine dysregulation promotes systemic low-grade inflammation, oxidative stress and endothelial dysfunction—all of which impair nitric oxide bioavailability and cavernosal smooth muscle relaxation [24]. Additionally, obesity is associated with hypothalamic-pituitary-gonadal axis suppression, leading to relative hypogonadism, reduced sex hormone-binding globulin and increased estrogen conversion via aromatase activity in adipose tissue [25]. The combination of endothelial dysfunction, chronic inflammation and hormonal alterations creates a synergistic negative effect on erectile function. Importantly, visceral adiposity—rather than body mass index alone—correlates more strongly with ED severity, underscoring the need for waist circumference measurement as a simple clinical tool [26]. Weight loss, even modest (5–10% of total body weight), has been shown to improve erectile function, reduce inflammatory markers and restore testosterone levels, making obesity one of the most treatable contributors to ED.

ED is conventionally classified into organic, psychogenic, iatrogenic and mixed types. The organic category is further subdivided into vasculogenic, neurogenic and endocrinologic etiologies [27]. Vasculogenic ED represents the most prevalent form, resulting from inadequate arterial inflow or excessive venous leakage and is frequently observed in conjunction with systemic atherosclerosis [28]. Neurogenic ED arises from disruption of central or peripheral neural pathways, commonly secondary to pelvic surgery, spinal cord injury or diabetic and non-diabetic neuropathies. Endocrinologic disorders—including hypogonadism, hyperprolactinemia, thyroid dysfunction and adrenal insufficiency—may compromise libido and erectile function through hormonal imbalance [29,30]. Contemporary consensus within the medical community acknowledges that psychogenic factors, once considered the predominant etiology, now represent one component within a complex interplay of multiple contributing factors [31]. The International Society for Sexual Medicine (ISSM) distinguishes between generalized psychogenic ED, associated with chronic anxiety, depression or diminished libido and situational forms linked to specific partners or contexts [32]. In clinical practice, organic and psychological causes frequently coexist, underscoring the necessity of comprehensive etiological evaluation to establish an accurate diagnosis. This is particularly pertinent in younger men, in whom ED may serve as an early indicator of underlying vascular or endocrine pathology [33]. While the central role of endothelial dysfunction and NO bioavailability is undisputed, the relative contribution of venous leakage versus arterial insufficiency in mixed vasculogenic ED remains debated, with direct implications for treatment selection [34].

From a clinical standpoint, what is established is that erectile function depends on a precise interplay between NO-cGMP signaling, cavernosal smooth muscle tone and pelvic floor musculature, with endothelial dysfunction representing the final common pathway in most organic ED cases. It is also well-established that ED frequently precedes coronary artery disease by two to five years, making it an essential cardiovascular sentinel [35].

What remains debated is the relative contribution of venous leakage versus arterial insufficiency in mixed vasculogenic ED, as well as the optimal threshold for diagnosing clinically significant veno-occlusive dysfunction. Additionally, whether pelvic floor rehabilitation should be prescribed as a standalone therapy or only as an adjunct remains unresolved.

For daily practice, clinicians should measure waist circumference in every man with ED, as central obesity—rather than BMI alone—is a treatable cause that correlates strongly with ED severity and hypogonadism. Furthermore, all men with unexplained ED warrant cardiovascular risk assessment, as erectile symptoms may be the earliest manifestation of systemic atherosclerosis.

## 4. Diagnosis and Evaluation

The diagnostic approach to ED begins with a comprehensive medical, sexual and psychosocial history. This foundational step is essential for identifying potential underlying etiologies and assessing the condition’s impact on the patient’s quality of life [36]. A detailed history enables the clinician to distinguish between lifelong and acquired ED, determine whether the dysfunction is generalized or situational and identify comorbid conditions that may contribute to or exacerbate the patient’s symptoms [37]. The incorporation of standardized assessment instruments, such as the International Index of Erectile Function (IIEF), facilitates objective quantification of ED severity, monitors therapeutic response and provides valuable patient-reported outcome measures [38,39]. The IIEF exists in two versions: the original 15-item IIEF (IIEF-15), which assesses five domains (erectile function, orgasmic function, sexual desire, intercourse satisfaction, overall satisfaction) and the abbreviated 5-item version (IIEF-5) commonly used as a brief screening tool in clinical practice [40]. Other self-report instruments, such as the Erectile Hardness Score (EHS) and the Sexual Encounter Profile (SEP), are also utilized to assess specific treatment outcomes. It is important to note that all such tools are self-reported, which may introduce recall or social desirability bias [41,42]. A thorough physical examination constitutes an indispensable component of the diagnostic evaluation, serving to identify signs of systemic disease or endocrine dysfunction [37]. Clinicians should assess body mass index, evaluate secondary sexual characteristics and perform a genital examination to detect penile deformities or testicular atrophy that may suggest hypogonadism. Waist circumference measurement is particularly important, as central obesity (defined as ≥94 cm in Caucasian men, with ethnic-specific cutoffs for other populations) correlates more strongly with ED severity and hypogonadism than BMI alone [43]. The presence of increased waist circumference should prompt targeted metabolic evaluation and aggressive lifestyle counseling, as this finding identifies men most likely to benefit from weight loss interventions. Assessment of blood pressure and cardiovascular status is mandatory, given the well-established association between ED and cardiovascular disease (CVD) [44,45]. Laboratory evaluation is essential for identifying the underlying causes of ED. Current guidelines recommend assessment of fasting glucose or HbA1c, lipid profile and morning total testosterone as initial tests [46]. Testosterone screening is particularly important in men with increased waist circumference or obesity, as these populations are at higher risk for hypogonadism [47]. Identifying hypogonadism before treatment is critical, as testosterone deficiency may require specific management [48]. When clinically indicated, additional assessment of prolactin and thyroid-stimulating hormone (TSH) may be performed to investigate endocrinopathies such as hyperprolactinemia or thyrotoxicosis [49,50]. Although fasting glucose, lipid profile and morning total testosterone are mandatory initial tests with strong guideline support, routine prolactin and thyroid screening without clinical suspicion remains controversial; current recommendations limit such testing to men with reduced libido, gynecomastia or young age without vascular risk factors [51,52]. In practice, a low threshold for prolactin measurement in young men with unexplained ED is prudent, given the possibility of a pituitary adenoma. Furthermore, considering the connection between ED and CVD, contemporary guidelines—including those from the European Association of Urology (EAU)—recommend cardiovascular risk assessment for all men presenting with ED, particularly those under 50 years of age, to evaluate for subclinical CVD [53,54]. Advanced diagnostic testing is reserved for select cases where initial evaluation remains inconclusive or when first-line therapy proves unsuccessful. Penile Doppler ultrasonography following intracavernosal administration of a vasoactive agent represents the gold standard for vascular assessment, enabling differentiation between arterial insufficiency and veno-occlusive dysfunction [55]. Standard hemodynamic parameters indicative of normal vascular function include peak systolic velocity exceeding 30 cm/s, end-diastolic velocity below 3 cm/s and a resistance index greater than 0.8. Demonstration of a normal flow pattern effectively precludes the need for further vascular investigation [56]. Nocturnal penile tumescence and rigidity (NPTR) testing serves as a valuable adjunct in discriminating between psychogenic and organic ED [57]. This modality records the occurrence, quality, duration and circumferential changes in nocturnal erections over a minimum of two consecutive nights using strain gauges. Preservation of normal nocturnal rigidity suggests intact vascular and neurologic function, thereby supporting a psychogenic etiology. Thus, NPTR monitoring proves particularly useful when psychogenic dysfunction is suspected [38]. Psychosexual assessment represents an integral component of comprehensive ED evaluation. Psychological distress, performance anxiety, depression and relational discord rank among the most prevalent factors contributing to erectile difficulties [58]. These elements may serve not only as primary etiological factors but also significantly influence treatment adherence and therapeutic outcomes. A structured psychosexual evaluation should address both individual and relational dimensions, with partner involvement encouraged whenever feasible. Emerging technological innovations, including artificial intelligence-based assessment platforms and virtual reality-enhanced simulations of psychosexual response, represent promising adjuncts to conventional diagnostic methods [59]. These approaches may enhance our understanding of real-world arousal patterns and patient behavior. Although these tools remain under investigation, they constitute an exciting frontier that may eventually complement established evaluation techniques [60].

What is established in the diagnostic workup is that a comprehensive medical, sexual and psychosocial history combined with standardized instruments such as the IIEF-5 provides objective quantification of ED severity. Mandatory initial laboratory tests—fasting glucose or HbA1c, lipid profile, and morning total testosterone—have strong guideline support.

What remains debated is whether routine penile Doppler ultrasound should be performed before empirical therapy or reserved for PDE5i non-responders, as no prospective trial has compared outcomes with versus without Doppler guidance. Similarly, routine prolactin and thyroid screening without clinical suspicion remains controversial; current guidelines limit such testing to men with reduced libido, gynecomastia, or young age without vascular risk factors.

For daily practice, a low threshold for prolactin measurement in young men with unexplained ED is prudent, given the possibility of a pituitary adenoma. Penile Doppler ultrasound should not be ordered routinely before first-line therapy; it is best reserved for cases where initial treatment fails or when considering invasive interventions. Waist circumference measurement should be performed in every patient, as central obesity identifies men most likely to benefit from targeted weight loss interventions.

## 5. Treatment and Management

**Figure 1:**
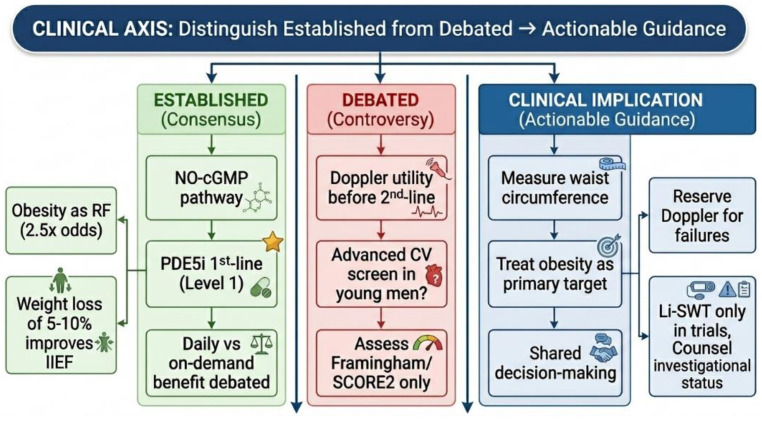
Clinical decision framework for ED management: established evidence, debated areas and practice implications.

Contemporary management of ED employs a patient-centered, stepwise therapeutic algorithm that accounts for disease etiology, severity and associated comorbidities. Multiple international guidelines—including those from the EAU, the American Urological Association (AUA) and the International Society for Sexual Medicine (ISSM)—support a sequential approach that begins with lifestyle optimization and risk factor modification, followed by pharmacologic intervention, then assistive devices and finally surgical options when indicated [61,62,63,64,65]. The 2025 EAU Guidelines emphasize individualized, shared decision-making, yet the overarching principle of progressing from least to most invasive interventions remains widely accepted. Each therapeutic decision must be individualized according to patient preference, psychosocial context and overall health status (Table 1) (Figure 1).

### 5.1. Lifestyle Optimization

Lifestyle modification constitutes the foundational intervention in ED management. ED is intrinsically linked to impaired endothelial function and reduced NO bioavailability; accordingly, dietary modification, physical activity and metabolic control have demonstrated significant therapeutic benefit [66,67]. Regular aerobic exercise, structured weight loss programs and smoking cessation promote vasodilation and enhance endothelial function. The Mediterranean diet—characterized by high consumption of fruits, vegetables, whole grains and unsaturated fats—has been associated with improved erectile function [68,69]. Weight loss deserves emphasis as a particularly effective intervention in men with obesity and ED. A meta-analysis of randomized controlled trials has demonstrated that lifestyle-induced weight reduction of 5–10% of total body weight significantly improves IIEF scores, with benefits correlating directly with the magnitude of weight loss and reduction in waist circumference [70]. More substantial weight loss achieved through bariatric surgery has shown even more pronounced effects, with some studies reporting normalization of erectile function in over 50% of obese men with ED at 12–24 months post-surgery [71,72]. The mechanisms underlying these improvements include enhanced endothelial function, reduced oxidative stress, increased testosterone levels and resolution of concomitant metabolic disturbances such as diabetes and dyslipidemia. Clinicians should therefore prioritize weight management as a first-line intervention in overweight and obese men with ED, recognizing that even modest weight reduction can produce meaningful clinical benefits. Additional lifestyle factors, including moderate alcohol consumption, adequate sleep and stress management, contribute to hormonal regulation and vascular health [73,74]. Conversely, poor dietary habits, sedentary behavior and substance dependence exacerbate endothelial dysfunction and oxidative stress, thereby potentiating ED severity. Adoption of healthy lifestyle modifications is efficacious not only in preventing ED but also in augmenting the therapeutic response to pharmacologic interventions [75].

### 5.2. Pharmacologic Therapy

Pharmacologic intervention remains the cornerstone of ED management. PDE5is—including sildenafil, tadalafil, vardenafil and avanafil—constitute first-line therapy. These agents enhance penile blood flow by inhibiting PDE5, thereby prolonging cGMP-mediated smooth muscle relaxation within the corpora cavernosa [76,77]. The distinct pharmacokinetic profiles of individual PDE5is (summarized in Table 2) allow for personalized regimens tailored to sexual habits and patient preferences [78,79]. Beyond enhancing erectile response, PDE5is facilitate recovery in both vascular and psychological domains. Daily tadalafil (5 mg) confers additional benefit in men with concomitant lower urinary tract symptoms secondary to benign prostatic hyperplasia (LUTS/BPH), addressing both conditions simultaneously and improving treatment adherence [80]. Consequently, PDE5is selection should be guided by a comprehensive assessment of patient health status, treatment preference and frequency of sexual activity. Regarding cardiovascular safety, extensive clinical experience and multiple randomized trials have not demonstrated increased risk of myocardial infarction or major adverse cardiovascular events associated with PDE5is therapy [54,81,82]. Beyond the absolute contraindication with organic nitrates, clinicians should be mindful of clinically significant drug–drug interactions. Concomitant use with alpha-blockers can potentiate hypotensive effects; therefore, co-administration should be managed with appropriate dose selection and staggering of dosing intervals to mitigate the risk of symptomatic hypotension. Additionally, potent inhibitors of cytochrome P450 3A4 (CYP3A4), such as ritonavir, ketoconazole and clarithromycin, can substantially increase plasma concentrations of PDE5is, necessitating dose adjustment or cautious use to avoid adverse events [83].

What is established is that PDE5 inhibitors represent first-line pharmacotherapy with Level 1 evidence supporting their efficacy and cardiovascular safety across diverse patient populations, provided organic nitrates are contraindicated. The superiority of PDE5is over placebo is undisputed, with 70–80% success rates in the general population.

What remains debated is whether daily tadalafil confers additional cardiovascular or endothelial benefits beyond on-demand dosing, or whether its principal advantage is limited to improved spontaneity and concomitant treatment of LUTS/BPH. Furthermore, whether combination therapy with testosterone benefits eugonadal men who are PDE5i non-responders lacks high-quality evidence.

For daily practice, daily tadalafil (5 mg) should be reserved for men who engage in frequent sexual activity (at least twice per week) or who have bothersome lower urinary tract symptoms. For men with high cardiovascular risk, initiation of sexual activity and PDE5i use should adhere to the Princeton Consensus Criteria, with collaboration between cardiology and sexual health specialists. When a patient reports PDE5i non-response, always verify adherence, on-demand dosing timing and adequate sexual stimulation before considering treatment escalation.

### 5.3. Second-Line Pharmacotherapy

Men who demonstrate non-response or intolerance to oral PDE5is may be considered for alternative pharmacotherapeutic options, including alprostadil. Alprostadil, a prostaglandin E1 analog, may be administered via intraurethral suppository or intracavernosal injection. As detailed in Table 1, intraurethral alprostadil offers a less invasive option with moderate efficacy, whereas intracavernosal alprostadil achieves higher success rates, particularly in men with diabetes or post-prostatectomy ED, but requires careful patient education to minimize risks such as priapism and penile fibrosis [84,85,86].

### 5.4. Mechanical Devices

Vacuum erection devices (VEDs) constitute a mechanical, non-pharmacologic alternative suitable for use as monotherapy or in combination with pharmacologic agents. VEDs generate negative pressure around the penis, inducing blood flow into the corpora cavernosa, which is subsequently maintained by a constriction ring applied to the penile base. Although reported success rates exceed 90%, some patients experience discomfort, ecchymosis or diminished spontaneity. VEDs are particularly advantageous for older adults, individuals with multiple comorbidities and patients preferring non-invasive management strategies [87,88].

### 5.5. Testosterone Replacement Therapies

In selected patients presenting with hypogonadism, diminished libido and inadequate response to PDE5is, testosterone replacement therapy (TRT) may be appropriate. Restoration of serum testosterone to physiologic levels can enhance sexual desire and erectile function, while concurrently improving mood, muscle mass and bone mineral density. TRT may be administered via intramuscular, transdermal or oral formulations, with selection guided by patient preference and tolerability. Comprehensive baseline evaluation and regular hormonal and hematologic monitoring, including hematocrit, prostate-specific antigen and testosterone levels at 3–6 month intervals during the first year of therapy, are essential components of safe and effective TRT administration [89,90].

### 5.6. Psychosocial Interventions

Psychosocial therapies constitute an integral component of multidisciplinary ED management. Cognitive-behavioral therapy (CBT), sex therapy and couples counseling assist patients in addressing maladaptive cognitive patterns, performance anxiety and interpersonal difficulties that frequently underlie or exacerbate ED. When combined with pharmacotherapy, psychological interventions enhance treatment adherence, promote sustained functional recovery and improve relational dynamics [91,92]. Although meta-analyses support the efficacy of psychological interventions, heterogeneity in treatment protocols and outcome measures limits the strength of pooled estimates; nonetheless, current evidence consistently favors a combined pharmacopsychosocial approach [93]. Psychotherapeutic support should be incorporated into urologic care, particularly for younger patients and those with psychogenic ED.

### 5.7. Surgical Management

For men with refractory ED or those desiring definitive treatment, surgical placement of a penile prosthesis represents the final intervention in the therapeutic algorithm. Available prostheses include inflatable and semi-rigid (malleable) devices, with selection based on patient anatomy and preference. Three-piece inflatable prostheses are most commonly selected due to their capacity to produce natural-appearing erections and superior concealability in the flaccid state [94].

What is established is that penile prosthesis implantation is the definitive treatment for refractory ED, with patient and partner satisfaction exceeding 95% when performed in experienced high-volume centers. With the adoption of no-touch surgical technique, antibiotic-coated devices and experienced surgical personnel, infection and mechanical failure rates have been reduced to below 5% [95,96,97].

What remains debated is the optimal timing of referral for prosthetic surgery—specifically, how many failed less invasive treatments should be attempted before considering a prosthesis—and whether routine penile Doppler ultrasound should guide the decision to proceed with implantation. Additionally, the comparative effectiveness of inflatable versus semi-rigid devices in specific patient populations remains an area of ongoing discussion.

For daily practice, referral for penile prosthesis implantation is appropriate when less invasive options have failed or are contraindicated, and when the patient desires a definitive, one-time solution. Patients should receive thorough preoperative counseling regarding risks, benefits, and realistic expectations, including the irreversible nature of the procedure. When the procedure is performed by an experienced surgeon, outcomes are excellent, and prosthesis implantation should not be viewed as a last resort but rather as a reliable, high-satisfaction option.

### 5.8. Regenerative Medicine

Emerging regenerative medicine approaches, including Li-SWT and PRP injections, represent novel therapeutic modalities with disease-modifying potential [98,99]. It is established that current evidence does not support routine clinical use; the 2025 EAU guidelines assign a weak recommendation to Li-SWT and no recommendation to PRP due to insufficient data [61]. What remains debated is whether these therapies offer any durable benefit beyond placebo. Optimal treatment protocols—energy levels, session frequency and number of treatments—have not been standardized and sham-controlled trials have yielded conflicting results [100,101]. No study has demonstrated improvement lasting beyond 12 months [102,103]. For clinical practice, these therapies should be discussed as investigational and offered only within clinical trials or after thorough counseling about uncertain long-term efficacy. Li-SWT induces mechanical microtrauma that activates angiogenic growth factors and progenitor cells, promoting neovascularization and endothelial repair. However, ongoing debate persists regarding optimal Li-SWT protocols—including energy levels, treatment frequency and number of sessions—which limits the comparability of clinical outcomes across studies. Clinical studies have demonstrated modest but consistent improvements in IIEF scores and erectile hardness measures, particularly in men with mild-to-moderate vasculogenic ED and those with suboptimal response to PDE5is [104,105]. Studies confirm modest efficacy, but significant heterogeneity in treatment protocols and sham controls precludes definitive conclusions regarding optimal patient selection and long-term durability [106,107].

PRP therapy involves intracavernosal injection of autologous platelets concentrated with vascular endothelial growth factor (VEGF), platelet-derived growth factor (PDGF), fibroblast growth factor (FGF) and insulin-like growth factor-1 (IGF-1), factors implicated in tissue regeneration and neovascularization [108]. Current evidence for PRP consists predominantly of small, non-randomized studies with short follow-up; thus, its efficacy remains unconfirmed and routine use is not recommended outside of clinical trials [109,110].

Despite encouraging preliminary findings, Li-SWT and PRP remain investigational, with ongoing efforts to establish standardized treatment protocols and demonstrate durable long-term efficacy. Why the evidence is not yet sufficient for routine clinical use requires explicit examination. First, conflicting study results abound, while several meta-analyses report modest IIEF improvements (2–4 points) with Li-SWT, multiple well-designed sham-controlled trials have shown no significant difference between active and sham treatment, suggesting a substantial placebo effect. Second, differences in treatment protocols are a major limitation—studies vary widely in energy flux density (0.05–0.30 mJ/mm^2^), number of shocks per session (1500–5000), number of sessions (4–12) and treatment intervals (weekly to biweekly), making direct comparison impossible and preventing identification of an optimal regimen. Third, problems with control groups are pervasive: many studies use inactive sham devices that patients can distinguish (lack of acoustic and tactile feedback), introducing performance bias; few employ rigorous blinding of both patients and outcome assessors. Fourth, long-term safety data are almost absent; no study has followed patients beyond 12–18 months, leaving unknown the potential for late adverse effects such as fibrosis, calcification or accelerated endothelial senescence from repeated mechanical trauma. Furthermore, PRP faces additional limitations: there is no standardization of preparation methods (centrifugation protocols, platelet concentration, activation status) and no randomized sham-controlled trial has demonstrated efficacy over placebo. Given these limitations—conflicting results, protocol heterogeneity, poor blinding and absent long-term safety data—current evidence supports the use of Li-SWT and PRP only in experimental settings or clinical trials. The 2025 EAU guidelines assign a weak recommendation to Li-SWT and no recommendation to PRP. Clinicians should counsel patients that these therapies remain investigational and out-of-pocket costs are not justified by current evidence.

What is established is that current evidence does not support the routine clinical use of Li-SWT or PRP injections for ED. The 2025 EAU guidelines assign a weak recommendation to Li-SWT and no recommendation to PRP due to insufficient data.

What remains debated is whether these therapies offer any durable benefit beyond placebo. Optimal treatment protocols—energy levels, session frequency, number of treatments—have not been standardized, and sham-controlled trials have yielded conflicting results. No study has demonstrated improvement lasting beyond 12–18 months, and long-term safety data are absent.

For daily practice, these therapies should be discussed as investigational and offered only within clinical trials or after thorough counseling about uncertain long-term efficacy. Patients should be informed that out-of-pocket costs are not justified by current evidence, and that neither therapy has been shown to modify the natural history of ED or to provide durable benefit beyond one year.

### 5.9. Integrative Model

**Figure 2:**
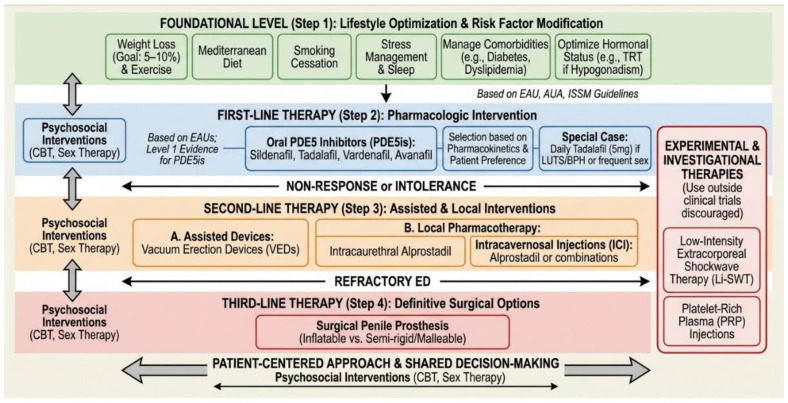
Integrative model for the treatment of ED.

**Figure 3:**
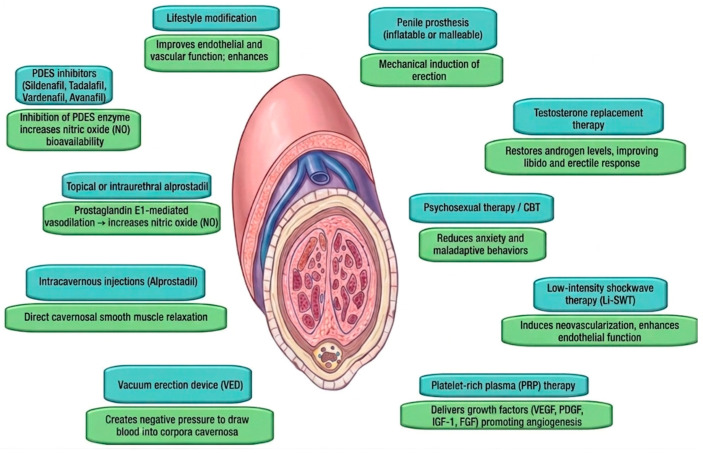
Comprehensive overview of treatment options for ED.

Contemporary ED management extends beyond the singular goal of restoring penile rigidity to encompass comprehensive sexual and psychological health recovery. The integration of lifestyle modification, pharmacologic and psychosexual therapy and regenerative or surgical approaches enables clinicians to develop treatment plans addressing both biological and relational dimensions of the condition. This integrative paradigm targets not only sexual function but also patient satisfaction, emotional well-being and quality of life—core objectives of modern andrology and sexual medicine [111,112] (Figure 2 and Figure 3).

### 5.10. Limitations of the Evidence and Persistent Gaps

Several limitations of the available evidence and persistent knowledge gaps should be acknowledged. First, while PDE5is are supported by Level 1 evidence, up to one-third of men demonstrate inadequate response and the mechanisms underlying primary non-response—including genetic polymorphisms, severe endothelial dysfunction and neurogenic factors—remain incompletely characterized [113]; standardized algorithms for managing non-responders are lacking. Second, for regenerative therapies (Li-SWT, PRP), the evidence base is limited by small sample sizes, heterogeneous treatment protocols (energy levels, session frequency, number of treatments), short follow-up durations (most ≤12 months) and poor blinding in many sham-controlled trials; no study has demonstrated durable benefit beyond 12–18 months and long-term safety data are absent. Third, meta-analyses of psychosexual interventions confirm efficacy, but substantial heterogeneity in treatment protocols, outcome measures and control conditions limits the precision of pooled estimates [114]. Fourth, the utility of routine penile Doppler ultrasound before second-line therapy remains debated, as no prospective trial has compared outcomes with versus without Doppler guidance [56,115]. Fifth, whether advanced cardiovascular screening (e.g., coronary CT angiography) benefits young men with ED and no cardiac symptoms is unknown. Sixth, most pharmacologic and device studies report outcomes only at 3–12 months, leaving a gap in understanding the long-term durability of response. Seventh, emerging digital health tools—telemedicine platforms, mobile applications and artificial intelligence-assisted assessment—lack validation in large prospective trials and concerns regarding data privacy and regulatory oversight persist [116,117]. These limitations do not invalidate the conclusions of this review but rather highlight priority areas for future research.

## 6. Discussion

ED remains a highly prevalent condition with significant medical and psychosocial implications. Although its incidence increases with age, recent epidemiological data have documented a rising frequency among younger men, frequently associated with unhealthy lifestyle behaviors, metabolic disturbances and stress-related disorders [118,119,120]. This shift in demographic distribution underscores the necessity of early diagnostic strategies and the integration of comprehensive men’s health assessments into preventive care, extending beyond the narrow scope of erectile function. In clinical practice, ED should be regarded as a potential systemic marker, often preceding the onset of cardiovascular disease, diabetes or endocrine dysfunction [121]. This paradigm has transformed the therapeutic approach from a symptom-centered model focused exclusively on penile rigidity to a more holistic evaluation of the patient, encompassing the interdependent vascular, endocrine and psychological domains. The introduction of PDE5is represented a transformative milestone in the pharmacological management of ED and these agents remain the cornerstone of treatment [122]. Their efficacy, safety profile and convenience of administration have substantially influenced both clinical practice and patient perceptions of sexual dysfunction [123]. However, variability in therapeutic response—particularly among individuals with diabetes, cardiovascular disease or a history of radical pelvic surgery—highlights the need for individualized treatment strategies [112,124,125]. Clinical experience and accumulating evidence indicate that multimodal approaches combining pharmacotherapy with psychosexual support and lifestyle modification yield superior outcomes compared to pharmacotherapy alone. Indeed, interventions targeting metabolic syndrome, obesity or sleep disturbances may substantially enhance erectile function while concurrently improving overall health outcomes. Although sometimes perceived as ancillary, these factors are critical to achieving sustained therapeutic success [126,127,128]. The relationship between obesity and ED warrants specific emphasis in clinical practice. Recent large-scale epidemiological studies have confirmed a dose–response relationship between increasing BMI and ED prevalence, with obese men having approximately 2.5-fold higher odds of ED compared to normal-weight peers [22,129]. Importantly, this association is partially reversible: a 2024 systematic review and meta-analysis of 22 studies involving over 3000 men demonstrated that weight loss interventions—ranging from dietary modification to bariatric surgery—consistently improved erectile function, with surgical weight loss producing the largest effects (mean IIEF improvement of 8–10 points) [130]. The clinical implication is clear: obesity should not be viewed merely as a comorbidity but as a primary therapeutic target.

Beyond these specific debates, several broader evidence gaps merit emphasis, including the lack of standardized algorithms for PDE5 inhibitor non-responders, the absence of long-term safety and efficacy data for regenerative therapies and the limited validation of digital health tools in prospective trials. Emerging regenerative modalities, such as Li-SWT and PRP injections, aim to restore physiological erectile mechanisms through endothelial repair and neovascularization [131]. Although the evidence base remains nascent, these approaches hold promise for modifying the natural history of ED, particularly in vasculogenic cases and PDE5is non-responders. However, the available literature is limited by small sample sizes, heterogeneous treatment protocols and a lack of long-term follow-up; consequently, the magnitude and durability of clinical benefit remain uncertain [109,110]. Nevertheless, their use remains investigational and standardized protocols are needed before they can be incorporated into routine practice [132,133]. Psychological and relational factors continue to exert a profound influence on the manifestation and perpetuation of ED [134]. The condition frequently coexists with anxiety, depression or interpersonal discord, creating a self-reinforcing cycle of psychological distress and sexual dysfunction. Incorporating cognitive-behavioral therapy (CBT) or couples counseling into treatment regimens can disrupt this cycle and facilitate the restoration of sexual confidence. Technological innovations—including teleconsultation, virtual reality-based rehabilitation and artificial intelligence-assisted assessment tools—are poised to further personalize patient care [135,136]. Surgical intervention, particularly penile prosthesis implantation, remains an essential component of the therapeutic algorithm for refractory ED. Despite its invasiveness, prosthesis implantation is associated with the highest satisfaction rates among patients and partners when preceded by appropriate counseling and executed with surgical precision [137,138]. Advances in device design, infection prophylaxis and minimally invasive surgical techniques have significantly improved outcomes, reinforcing the role of prosthetic surgery as a definitive and reliable option in appropriately selected candidates. From a broader perspective, contemporary management of ED should not be confined to the restoration of penile rigidity, but rather oriented toward the comprehensive recovery of sexual health as an integral dimension of quality of life. This objective necessitates a multidisciplinary collaborative framework involving urologists, endocrinologists, cardiologists, psychologists and rehabilitation specialists [139,140]. The future of ED management will likely be characterized by the integration of targeted pharmacotherapy, regenerative approaches and digital health platforms within a cohesive, patient-centered care pathway [104,141,142,143,144]. By synthesizing traditional clinical expertise with innovative therapeutic strategies, practitioners can transform the management of ED from a reactive intervention into a restorative process—one aimed not merely at treating dysfunction, but at rebuilding confidence, intimacy and overall male well-being.

Several key areas remain actively debated. First, whether young men with ED and no cardiac symptoms should undergo advanced cardiovascular screening (e.g., coronary CT angiography) is controversial; current consensus recommends only risk factor assessment using Framingham or SCORE2, reserving advanced imaging for those with additional warning signs. Second, the utility of routine penile Doppler ultrasound before second-line therapy is contested: some experts advocate it to distinguish arterial from venogenic causes, while guidelines reserve it for patients who fail empirical therapy. Third, whether the combination of PDE5is and testosterone benefits eugonadal men with PDE5is non-response lacks high-quality evidence. Fourth, regenerative therapies remain highly controversial despite commercial availability. For each debate, we offer practical guidance: assess cardiovascular risk rather than image; consider Doppler only after treatment failure; check morning testosterone before labeling non-response and discuss regenerative options as investigational.

## 7. Conclusions

This review was organized around a single clinical axis: distinguishing what is established from what remains debated in ED management and translating both into actionable guidance for daily practice. ED is a multifactorial condition that often precedes cardiovascular disease by 2–5 years, making it an essential sentinel for preventive care. PDE5 inhibitors remain highly effective first-line therapy with Level 1 evidence supporting their safety and efficacy. Lifestyle optimization—particularly weight loss of 5–10% in obese men—produces meaningful improvements in erectile function. For refractory cases, penile prosthesis surgery achieves >95% patient satisfaction in experienced centers.

The utility of routine penile Doppler ultrasound before second-line therapy, the need for advanced cardiovascular screening in young men with ED, and whether daily tadalafil confers benefits beyond convenience and LUTS treatment. Regenerative therapies (Li-SWT and PRP) remain investigational; current evidence does not support routine clinical use.

Clinicians should measure waist circumference in every man with ED, as central obesity is a treatable cause. PDE5 inhibitors should be prescribed as first-line therapy, but daily tadalafil should be reserved for men who engage in frequent sexual activity (at least twice per week) or who have concomitant lower urinary tract symptoms secondary to benign prostatic hyperplasia. Penile Doppler ultrasound should not be ordered routinely before empirical therapy; it is best reserved for cases where initial treatment fails. Regenerative therapies such as low-intensity shockwave therapy and platelet-rich plasma injections should be discussed as investigational, and patients should be informed that out-of-pocket costs are not justified by current evidence. When less invasive options fail, referral for penile prosthesis implantation is appropriate, as outcomes are excellent when the procedure is performed by experienced surgeons.

Future research should focus on standardized algorithms for PDE5 inhibitor non-responders, long-term safety data for regenerative modalities, and validation of digital health tools in prospective trials.

## Figures and Tables

**Table 1:** Current treatment options for ED—synthesized evidence from EAU, AUA and ISSM guidelines.

Therapeutic Level	Intervention	Mechanism of Action	Advantages	Limitations/Adverse Effects	Success Rates/Key Evidence	Patient Types/Indications	Clinical Notes (Including Evidence Gaps)
First-line	Lifestyle modification (diet, exercise, smoking cessation)	Improves endothelial and vascular function; enhances NO bioavailability	Addresses modifiable risk factors; cardiovascular and metabolic benefits	Requires adherence and long-term commitment	IIEF improvement: 2–5 points; weight loss of 5–10% body weight, effective	All patients	Recommended for all patients; evidence gap: optimal duration and intensity of exercise not defined.
	PDE5is (Sildenafil, Tadalafil, Vardenafil, Avanafil)	Inhibition of the PDE5 enzyme increases cGMP → cavernosal smooth muscle relaxation	High efficacy, safety and non-invasiveness	Headache, flushing, dyspepsia; contraindicated with nitrates	70–80% success in the general population; lower in diabetes or post-prostatectomy	General ED population; daily tadalafil for men with LUTS/BPH or frequent sexual activity	First-line pharmacologic therapy; evidence gap: standardized algorithm for non-responders lacking
Second-line	Topical or intraurethral alprostadil	Prostaglandin E1-mediated vasodilation	Effective in PDE5is non-responders; localized action	Penile erythema, burning, transient pain	30–50% success for intraurethral	PDE5i non-responders, men preferring a less invasive alternative	Patient-specific option; evidence gap: long-term adherence data are sparse
	Intracavernous injections (Alprostadil)	Direct cavernosal smooth muscle relaxation	High efficacy across ED etiologies	Penile pain, priapism, fibrosis	70–85% success	PDE5i non-responders, diabetic or post-prostatectomy ED	Requires patient training; evidence gap: comparative efficacy vs. combination therapy unknown
Third-line	Vacuum erection device (VED)	Negative pressure draws blood into the corpora	Non-invasive; effective in up to 90%	Discomfort, bruising, reduced spontaneity	80–90% success for achieving erection; satisfaction ~60–70%	Older patients, multiple comorbidities, patients avoiding drugs/surgery	Suitable for selected patients; evidence gap: few RCTs, long-term dropout rates are high
	Penile prosthesis (inflatable or malleable)	Mechanical induction of erection	Definitive solution; high satisfaction	Surgical risks, infection and mechanical failure	≥90–95% patient/partner satisfaction; infection <5% in experienced centers	Refractory ED, failed all other therapies, penile fibrosis or Peyronie’s	Requires an experienced surgeon; evidence gap: comparative effectiveness of different device types
Adjunctive	Testosterone replacement therapy	Restores androgen levels, improving libido and erectile response	Effective in men with low or low-normal testosterone	Requires hormonal monitoring	Modest IIEF improvement (2–4 points) when combined with PDE5is in hypogonadal men	Hypogonadal men (morning testosterone <12 nmol/L) with ED	Combine with PDE5is; evidence gap: benefit in eugonadal PDE5i non-responders unclear
	Psychosexual therapy/CBT	Reduces anxiety and maladaptive behaviors	Improves sexual satisfaction and adherence	Needs trained specialists	Effect size moderate; combined with PDE5is, superior to PDE5is alone	Psychogenic or mixed ED, performance anxiety, relationship issues	Integral part of multidisciplinary care; evidence gap: optimal number and format of sessions
Emerging/Regenerative	Low-intensity shockwave therapy (Li-SWT)	Induces neovascularization, enhances endothelial function	Non-invasive, potential restorative effect	Still experimental; benefit modest but consistent	IIEF improvement 2–5 points vs sham; no durability >12 months	Vasculogenic ED, PDE5i non-responders (investigational)	Weak recommendation; evidence gap: optimal protocol, long-term safety, sham-controlled durability
	Platelet-rich plasma (PRP) therapy	Delivers growth factors (VEGF, PDGF, IGF-1, FGF), promoting angiogenesis	Potentially disease-modifying	Limited evidence; variable protocols	No RCT showing efficacy over placebo; small studies show minimal IIEF change	Not recommended for routine use; only in clinical trials	No recommendation; evidence gap: standardization, randomized sham-controlled trials, long-term outcomes

Inhibition of the PDE5 enzyme increases cGMP, leading to cavernosal smooth muscle relaxation.

**Table 2:** Summary of PDE5is for the treatment of ED.

Drug	Typical Dose and Administration	Onset of Action	Duration	Key Features/Notes
Sildenafil	25–100 mg orally, on demand (initial dose 50 mg)	30–60 min	Up to 12 h	Most studied PDE5is; Orally disintegrating tablets (ODT) formulation available for dysphagia
Tadalafil	10–20 mg on demand or 5 mg once daily	~30 min	Up to 36 h	Also improves LUTS/BPH; suitable for daily use and spontaneous activity
Vardenafil	5–20 mg on demand	~30 min (some effect after 15 min)	8–12 h	Available as ODT; similar efficacy to other PDE5is
Avanafil	50–200 mg on demand	15–30 min	6–8 h	Fastest onset; good tolerability; comparable efficacy

## Data Availability

Data sharing does not apply to this article as no datasets were generated or analyzed.
